# Thiopental sodium loaded solid lipid nano-particles attenuates obesity-induced cardiac dysfunction and cardiac hypertrophy via inactivation of inflammatory pathway

**DOI:** 10.1080/10717544.2020.1803449

**Published:** 2020-08-07

**Authors:** Canzhan Zhu, Wanjing Li, Xinhong Wang, Jiahong Xue, Ling Zhao, Yafan Song, Tian Zhou, Mingjuan Zhang

**Affiliations:** Department of Cardiology, The Second Affiliated Hospital of Xi’an Jiaotong University, No. 157 Xiwu Road, Xincheng District, Xi’an, Shaanxi, 710004, China

**Keywords:** Solid lipid nano-particles, cardiac dysfunction, thiopental sodium, cardiac remodeling, endothelial dysfunction, oxidative stress, inflammation

## Abstract

This work evaluates solid lipid nanoparticles of thiopental sodium against obesity-induced cardiac dysfunction and hypertrophy and explores the possible mechanism of action. TS loaded SLNs were formulated by hot-homogenization and solvent diffusion method. TS-SLNs were scrutinized for entrapment efficiency, drug loading capacity, gastric stability, particle size, in vitro drug release. Mice were feed with the normal chow or high-fat diet for 08 weeks to induce obesity and primary cardiomyocytes. The therapeutic effects of thiopental sodium in the high fat diet (HFD) induced cardiac hypertrophy. Systolic blood pressure (SBP) was estimated at a regular time interval. At the end of the experimental study, systolic pressure left ventricular, LV end-diastolic pressure and rate of increase of LV pressure and antioxidant, apoptosis, cytokines and inflammatory scrutinized. HFD induced group mice exhibited a reduction in the body weight and enhancement of cardiac hypertrophy marker and dose-dependent treatment of thiopental sodium up-regulation the body weight and down-regulated the cardiac hypertrophy. Thiopental sodium significantly (*p* < .001) dose-dependently altered the antioxidant, biochemical, cardiac parameters and remodeling. Thiopental sodium significantly (*p* < .001) dose-dependently reduced the SBP. Thiopental sodium altered the apoptosis marker, pro-inflammatory cytokines, inflammatory parameters along with reduced the p38-MAPK level. The cardiac protective effect of thiopental sodium shed light on future therapeutic interventions in obesity and related cardiovascular complications via inflammatory pathway.

## 1. Introduction

Disease-related death throughout Worldwide increases day by day and cardiovascular disease (CVD) related death ranked first among all types of disease. Previous literature suggests that cardiac hypertrophy is an ordinary response of the heart to various CVD stimuli such as hypertension, coronary heart disease, cardiomyopathy, valvular heart disease and hypertrophy (D’Agostino et al., 2008; Sumithran & Proietto, 2014). It is well documented that cardiac hypertrophy is boosting the mass of ancillary and contractile proteins of the heart (Shimizu & Minamino, 2016). In the initial stage, Cardiac hypertrophy is a compensatory response to normal, continuous cardiac function; but in the later stages, maladaptation results in pathological cardiac hypotrophy progression, which eventually leads to heart failure (Rosca et al., 2013; Lyon et al., 2015). Previous studies suggests that cardiac hypertrophy has played an important risk factor for adverse cardiovascular disease (Rosca et al., 2013; Lyon et al., 2015). Various factors such as oxidative stress and inflammation involved in the pathophysiologically of cardiac hypertrophy. Among the cardiovascular disease, obesity plays a significant role in the cardiac function and remodeling in terms of impaired ventricular contractility, myocardial fibrosis and hemodynamic load, which further lead the expansion of heart failure and heart disease (Dai et al., 2011; Rosca et al., 2013; Lyon et al., 2015). Previous research suggests that obesity has been described as an inflammatory state and oxidative stress and chronic inflammation play an imperative role in the pathogenesis of obesity and related complications (Abel & Doenst, 2011; Rosca et al., 2013). It has been confirmed that the, during the obesity enhance the level of fatty acid, which further activated the nuclear factor kappa B (NF-kB) pathway, afterward, boost the expression of inflammatory cytokines and reaction and inducing the oxidative stress (Silambarasan et al., 2014; Wang et al., 2019). Still, the molecular mechanisms involved in obesity-related cardiac hypertrophy still unclear.

The mitochondria are the powerhouse of the body, which involve in the production of ATP and occupy the 30–40% cell volume in the mammalian cardiomyocytes in adult. Pre-clinical and clinical investigation suggests that mitochondrial dysfunction contributes to the expansion of cardiac hypertrophy.

It is well documented that cardiac hypertrophy is the risk factor for adverse CVD events. Various factors are involved in the pathophysiology of cardiac hypertrophy such as oxidative stress and inflammation plays a crucial role in the expansion of disease (Silambarasan et al., 2014; Wang et al., 2019). Among cardiovascular disease, obesity has a great impact on cardiac remodeling and its function such as myocardial fibrosis, damaged ventricular contractility and hemodynamic load, which leads to the expansion of heart failure (Mooney, 2012). Previous research suggests that obesity categorized as an inflammatory reaction and oxidative stress and low-grade inflammation play a significant role in the pathogenesis of obesity-linked complications (Ferrucci & Fabbri, 2018). It is well proved that nuclear factor-kappa B (NF-kB) pathway activated during the cardiac disease, which further enhances the oxidative stress and various pro-inflammatory cytokines (Siti et al., 2015; Ferrucci & Fabbri, 2018). Therefore, the possible molecular mechanism involved in obesity-induced cardiac hypertrophy still unclear. Hence, searching for the new therapeutic drug such as thiopental sodium for the treatment of cardiac hypertrophy, which minimizes or reverse the progression rather than treating the vested heart failure or cardiomyopathy, is the current strategy.

Thiopental sodium is a lipid soluble anesthetic. Which decrease the level of endogenous dopamine, adrenaline and nor-adrenaline. According to Ebert et al., showed that thiopental sodium shows the bronchospasm by suppressing the sympathetic activity (Ebert et al., 1990). Thiopental sodium also exhibited antioxidant effect by decreasing the lipid peroxidation (LPO) or by suppressing ROS production by neutrophils (Dogan et al., 2010). Thiopental sodium exhibited the lesser degree of anti-hemolytic effect by reducing the free radical attributed hemolysis of red blood cell (ROS) during the *in vitro* activity (Nishina et al., 1998). According to Kobayashi et al., thiopental sodium exhibited neuro-protective effect against the on brain ischemia via estimation of the ischemic time necessary for inducing the 50% neuronal damage in hippocampus CA1 regions (Kobayashi et al., 2007). Due to the anti-inflammatory and antioxidant effect of thiopental sodium, in this experimental study, we scrutinized the protective effect of solid lipid nanoparticle of thiopental sodium against Obesity-induced Cardiac dysfunction and Cardiac hypertrophy via inactivation of inflammatory and oxidative pathway.

## 2. Material and methods

### 2.1. Preparation of the Thiopental sodium loaded SLNs (TS-SLNs)

The TS loaded SLNs were formulated by hot-homogenization and solvent diffusion method as stated throughout literature reports with suitable modifications (Ekambaram & Abdul Hasan Sathali, 2011; Rahman et al., 2019). The selection of the amount of solid lipid used for preparing SLNs was selected based on solubility study. In this respect, Compritol 888 ATO (i.e. 250 mg) and Phospholipid 90 G (PL90G) (60 mg) were chosen as the solid lipid and co-surfactant, and both were heated up to 70 °C. A fixed quantity of the medication (i.e. 2.5 mg/kg of body weight) was applied with keep gentle mixing for full solubilization in the lipidic liquid. Still, 3% w/v of aqueous solution of Tween 80 was prepared in 10 mL of distilled water at 70 °C (Ekambaram & Abdul Hasan Sathali, 2011). Then aqueous phase was introduced into the organic phase under continuous homogenization at the speed of 10,000 rpm for 2–6 min to get a uniform dispersion. In addition, excess water (10 mL) was added to solvents and subsequently constantly stirred at 1600 rpm for 1–4 h in the ice bath state for the acquisition of SLNs and further the collected formulation deposited at 4 °C in the refrigerator (Ekambaram & Abdul Hasan Sathali, 2011).

### 2.2. Characterization of the TS-SLNs

#### 2.2.1. Particle size (PS)

The particle size distribution of TS-SLNS was obtained using of Dynamic light dispersion technique with Zetasizer ZS 90 (M/s Malvern Instruments, Worcestershire, UK).

#### 2.2.2. Entrapment efficiency (EE) and drug loading capacity (LC)

The EE and LC are known as the proportion of drug effectively stuck in the SLNs. It was determined by a dialysis technique according to the reported method (Rahman et al., 2019). Briefly, a SLN dispersion aliquot (2 mL) was centrifuged at 10,000 rpm (5590 ×g), supernatant was discarded and pallet was obtained. In addition, for digestion in 0.1% w/v solution of ethanol, the pellet comprising SLNs was applied for ultrasonication at 9 mV for 15 min for complete removal of the drug from the SLN. The drug was collected in mobile phases and quantified by HPLC. Equations 1 and 2 have been used as following for encapsulation efficiency and loading capacity.

(1) $$EE\%=\frac{\text{Total quantity of TS}-\text{Free quantity of TS}}{\text{Total Quantity of TS}}\times 100$$

(2) $$\text{LC }(\%)=\frac{\text{Total amount of TS encapsulated in SLN}}{\text{Total amount of SLN weight}}\times 100$$

#### 2.2.3. Transmission electron microscopy (TEM)

Aliquot 1-mL SLNs was diluted 100 times with triple distilled water and sprayed on a copper grid with 1% phosphotungstic acid (PTA) solution. The sample was allowed under the transmission electron microscope (JEM-2100F, M/s Jeol, Tokyo, Japan) to dry on board.

#### 2.2.4. In vitro *drug release study*

In vitro drug release study was performed for testing the release profile of the SLNs in 250 mL in phosphate buffer (pH 7.4), at 100 rpm and 37 ± 0.5 °C temperature for 24 h. The formulations are packed in the dialysis bag with molecular cutoff weight of 12 Da (M/s Himedia limited, Mumbai, India). SLN dispersion equal to 2.5-mg drug was loaded in the dialysis bag and subject to study. Aliquot (0.5 mL) of samples were taken regularly and loaded with the same quantity of fresh medium held at 37 ± 0.5 °C. The samples were analyzed by HPLC and cumulative percent drug release was determined. The obtained drug release data were fitted with various mathematical models such as zero-order, first-order, Higuchi and Korsmeyer – Peppas to evaluate the release kinetic based on comparison of the values of correlation coefficient (*R*). Additionally, the process of drug release from the SLNs was tested using release exponent (*n*) obtained from the Korsmeyer – Peppas equation (Rahman et al., 2019).

### 2.3. Stability studies

Binderfi KBF-240 temperature chamber was used for (Binder GmbHfi Ltd., Munchen, Germany) testing the performance of the optimized TS-SLN (Üner, 2006). The stability studies were conducted at 25 °C/60% RH and 40 °C/75%RH. The formulations were packed in the vials, tightly sealed and subjected to stability studies for 0, 1, 2, 4, 8 and 12 weeks. Furthermore, these formulations are tested for various characters such as PS, PDI, EE and LC at aforementioned time intervals.

### 2.4. Experimental animal

C57BL/6 mice (gender- male; age-8–10 weeks; body weight 22 ± 5 °C) were used for the current experimental protocol. The mice were received from the animal facility and kept in the standardized conditions such as kept in the polypropylene cage; temperature 20 ± 2 °C; relative humidity 35–65% and 12-h dark/12-h light cycle. The mice were received the commercial chew *ad libitum*. The whole excremental protocol was performed according to following the following and used in strict accordance with the rules and regulations outlined in the Guide for the Care and Use of Laboratory Animal issues via US National Institute of Health. The whole experimental protocol was approved by the Institutional animal ethical committee. Normal control mice revieved the normal diet (15 kJ/g, 14% energy as fat). High fat diet (22 kJ/g, with 60% of energy) was used for induction the obesity. The mice were received the HFD for 20 weeks. The HFD (lard based) were received from the Nanjing Junke Biotechnology Corporation, Ltd (Nanjing, China).

### 2.5. Experimental protocol

The mice were randomly divided into the six groups and each group contains the 10 mice. The mice were grouped as follows:

- Group A: control
- Group B: model control
- Group C: low dose of Thiopental sodium
- Group D: high dose of Thiopental sodium
- Group E: intermediate dose of Thiopental sodium loaded SLN
- Group F: treated with Fosinopril

Treated rats received the intragastric administration of thiopental sodium (1.25, 2.5 and 5 mg/kg, body weight) and fosinopril (2 mg/kg, body weight) from the start of week 5. Afterward, the mice were euthanized using the intraperitoneal injection of pentobarbital (200 mg/kg). At the end of the experimental study (8 weeks) the serum and heart tissue were sampled.

### 2.6. Estimation of systolic blood pressure

Tail cuff method was used for the estimation of systolic blood pressure (SBP) (ALC-NIBP; Shanghai Alcott Biotech Co., Ltd., Shanghai, China). Briefly, the basal BP was estimated in each group once before the experimental study and continued each week until the 8 weeks. All the SBL were performed at the same time of the day and performed by the same person. Before the estimation, the mice were warmed at 27 °C for 30 min to allow the measurement of pulsations of the tail artery to achieve a steady pulse level. The systolic blood pressures were estimated as the average time of 20.

### 2.7. Hemodynamics

For the estimation of hemodynamics, all groups of experimental mice were anesthetized using the chloral hydrate and the right carotid artery was cannulated using the catheter, which is connected to an admittance control unit. After that, the catheter was successfully inserted into the left ventricle by using the right coronary artery and four-channel acquisition systems were used for recording the signal. Before, the insertion of the catheters, it should be soaked into the Alconox for 30 min. The rate of LV pressure rise, LV end-diastolic pressure (LVEDP) and LV systolic pressure (LVSP) were recorded (Wang et al., 2019).

### 2.8. Collection of serum

For the estimation of the hemodynamic index, blood samples directly from the heart and kept in the heparin pretreated tubes and centrifuged for 10 min at the 35 g rpm at the 4 °C and the supernatant was stored at the −80 °C for further analysis (Lang et al., 2018).

### 2.9. Collection of cardiac tissues

At the end of the experimental study, all group mice were sacrificed and the heart tissue were successfully removed and calculated the heart to body weight ratio (HW/BW) by dividing the heart weight (HW) via body weight (BW) after the harvest and weighting the hearts (Lang et al., 2018; Wang et al., 2019).

### 2.10. Isolation of mitochondria

For isolation of mitochondria from the heart tissue, briefly, the heart left ventricle was cut into small fragments and homogenized by using the glass homogenizer and commercial kits were used for the isolation of mitochondria by following the manufacture instruction (Beyotime Institute of Biotechnology, Shanghai, China). After successful isolation of mitochondria, it is suspended into the MiR05 and immediately estimates the respiratory parameters by using the protein assay kits following the manufacture instruction (Beyotime Institute of Biotechnology, Shanghai, China).

### 2.11. Estimation of mitochondria parameters

Briefly, for the estimation of cardiac mitochondria respiratory rate by using the Oxygraph-2k at 37 °C and for the estimation of respiratory rates Datlab software was used. Mitochondrion (1 mg) was mixed into each chamber after respiration stabilization and after that, the 5 mM glutamate and substrates (2-mM malate) were added for the estimation of leak respiration of complex I (CI LEAK). 5 mM ADP was added for the estimation of oxidative phosphorylation of CI (CI OXPHOS) and 10 μM cytochrome C was mixed for the determination of outer membrane integrity of mitochondria. For the estimation, the oxidative phosphorylation capacity of CI + II (CI + II OXPHOS, state P) 2.5 mM succinate was added. Further, 1 μg/ml oligomycin added after the CI + II OXPHOS estimation. After that, the ability of electron transport chain was titrated after the addition of FCCP and finally, the CII respiration was scrutinized after mixed the 0.5 μM rotenone (CI inhibitor), by suppressing the respiration complex I. for the estimation the consumption of residual oxygen via CIII inhibition with the 2.5 μM antimycin A.

### 2.12. Estimation of ATP level

Firefly luciferase-based ATP assay kit was used for the determination of adenosine 5′-triphosphate (ATP) in the cardiac mitochondrial by following the manufacture instruction (Beyotime Institute of Biotechnology, Shanghai, China).

### 2.13. Endurance capacity

The endurance test was performed for the estimation of enhancing workload capacity. Briefly, before the experimental study, all the experimental mice were trained to run on the treadmill at a speed of 10 m/min and inclination at 0 for 15 min each day. For the study, the treadmill was set to a 10^°^ incline and has been exhibited to elicit maximum consumption O_2_. During the experimental study, all the mice forced to run for 10 min (10 m/min), 5 min (14 m/min) and till exhaustion (18 m/min). The exhaustion was mentioned as the failure to maintain the workload and remain on the electrical grid for 4 s.

### 2.14. Estimation of biochemical parameters

The previously reported method was used for the estimation of NO activity. Briefly, the NO level was rapidly changed to nitrite and nitrate and the NO kits were used for the estimation of nitrite and nitrate content in the serum (Nanjing Jiancheng Bio-Engineering, Nanjing, China) by using the manufacture’s instructions. L-arginine (L-Arg), Endothelin-1 (ET-1) and nitric oxide (NO) activity was estimated in the cardiac tissue by using the available EISA kits, following the manufacture instruction (Beyotime Institute of Biotechnology, Shanghai, China).

### 2.15. Estimation of antioxidant enzymes

Catalase (CAT), superoxide dismutase (SOD) and glutathione (GSH) were estimated using the EILSA kits (Nanjing Jiancheng Bio-Engineering, Nanjing, China) by using the manufacture instructions. Malonaldehyde (MDA), the level was estimated using the thiobarbituric acid reactive substances assay by using the standard available kits (Nanjing Jiancheng Bio-Engineering, Nanjing, China) using the manufacture instructions.

### 2.16. Pro-inflammatory cytokines

The level of pro-inflammatory cytokines such as IL-1β, IL-6, and TNF-α was determined into the heart tissue by using the ELISA kits (Excell Biotechnology, Shanghai, China).

### 2.17. Apoptosis marker

Apoptosis parameters such as caspase-3, caspase-8 and caspase-9 were estimated using the standard available ELISA kits following the manufacture instruction (Excell Biotechnology, Shanghai, China).

### 2.18. Statistical analysis

Graph Pad Prism software was used for the analyzed the data and whole the data showed as the mean ± standard deviation (SD). One way analysis of variance (ANOVA) followed via Dennett's test to compare the difference among the groups. *p* < .05, *p* < .01 and *p* < .001 are considered as significant, more significant and extremely significant.

## 3. Result

### 3.1. Selection of the ingredients

TS equilibrium solubility studies found solubility in different lipids in the following order: Compritol 888 ATO > GMS > Capmul MCM C10 >Stearic acid. Therefore, Compritol 888 ATO was selected as the lipid for formulation development. The equilibrium solubility of drug in surfactants and co-surfactantswas found to be in the order of Tween 80 > Unitop100 > Poloxamer 188and Phospholipid 90 G (PL90G) >Phospholipid 90H (PL90H) >lecithin soy. Therefore, Compritol 888 ATO, Tween 80 and Phospholipid 90 G (PL90G)were selected as the key ingredients for the formulation development of SLNs.

### 3.2. Characterization of the TS-SLNs

#### 3.2.1. Particle size

The particle size of TS-SLNs ranged between 50 and 110 nm, showing the formulation’s nanostructured nature. However, smaller particle size was obtained at medium lipid, surfactant, cosurfactant, fast homogenization speed and stirring speed, and vice versa.

**Figure 1.**
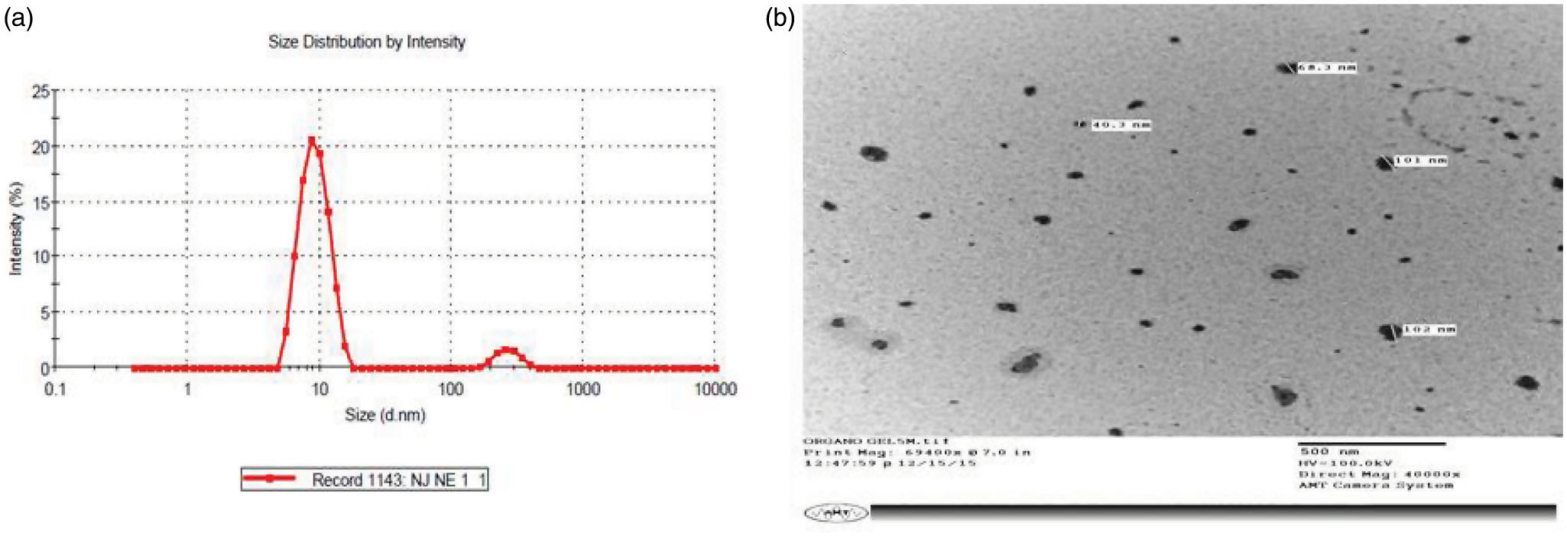
(A,B) Optimized TS-SLN characterization: (A) shows particle size and (B) TEM analysis.

Figure 1(A) illustrates the particle size distribution of optimized TS-SLN with value of 68.07 nm and polydispersity index (PDI) value of 0.149, while Figure 1(B) illustrates photographs of optimized TS-SLN transmission electron microscopy in nanosized form and spherical shape.

#### 3.2.2. Entrapment efficiency (EE) and drug loading capacity (LC)

The developed formulations showed high EEof 88% and LC of 13.3%.

#### 3.2.3. In vitro *drug release studies*

**Figure 2.**
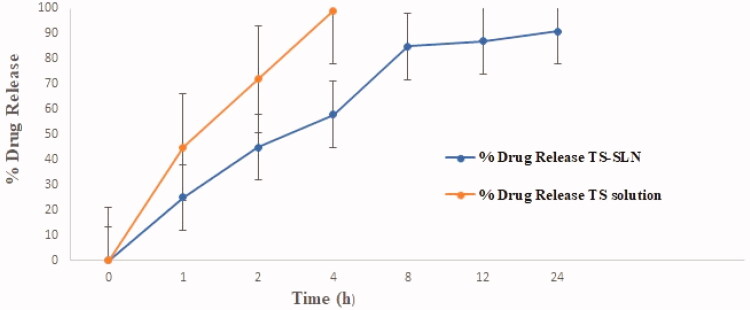
Thiopental sodium (TS), *in vitro* release from solid lipid nanoparticles SLN) and TS- solution at different time intervals (0–24 h) in phosphate buffer (pH 7.4) with dialysis method.

Figure 2 shows the SLN formulations in vitro drug release profiles of the modified TS-SLNs and TS solution. Different drug release characteristics were observed from these formulations with maximal drug release (i.e. >58%) within the first 4 h time span. Further, the data indicated that TS release from the SLNs exhibited a biphasic pattern of drug release within 4 h, while the remaining amount of drug release was observed within the next 10 h. The mathematical modeling of the release data from TS-SLN followed first-order release kinetics exhibiting an *R* value of 0.9651. Furthermore, the Korsmeyer – Peppas model applied to the drug release data showed that the release exponent (*n*) value for the optimized SLN is 0.821, thus suggesting drug release through anomalous (non-Fickian) diffusion mechanism. The reported findings are agreement with previously published studies on multiple reports (Üner, 2006; Ekambaram & Abdul Hasan Sathali, 2011).

### 3.3. Stability studies

#### 3.3.1. Particle size distribution and polydispersity index (PDI)

The optimized TS-SLN particle size (PS) stored at 25 °C/60% RH and 40 °C/75% RH ranged from 68 nm to 66 nm and 68.07 nm to 107.1 nm respectively (shown in Tables 1 and 2). During storage at 25 °C/60% RH, the PS of SLNs was unchanged, while at 40 °C/75% RH the particle size was increased due to the aggregation phenomenon due to an increase in system kinetic energy (Nayak et al., 2010). Nevertheless, the PDI data revealed a low and steady value, thereby at 25 °C/60% RH, demonstrating the physically stable existence of the PS for at least 12 weeks (as shown in Table 1).

**Table 1.** Particle size, entrapment efficiency and loading capacity of SLN after storage at 25°C/60%RH.

Time (in weeks)	Particle size (nm)	PDI	EE (%)	LC (%)
0	68.07	0.149	88	13.3
1	68	0.151	87.3	13.1
2	67.8	0. 23	87	12.8
4	67.5	0. 25	86.8	12.5
8	67	0.29	86.5	12.3
12	66	0. 33	86.4	12.2

SLN: Solid lipid nanoparticles; EE: Entrapment Efficiency; LC: Loading Capacity; PDI: Polydispersity Index.

**Table 2.** Particle size, entrapment efficiency and loading capacity of SLN after storage at 40°C/75%RH.

Time (in weeks)	Particle size (nm)	PDI	EE (%)	LC (%)
0	68.07	0.149	88	13.3
1	70.12	0.154	87.2	13.1
2	72.04	0.24	83.1	12.7
4	78.11	0.37	80.31	12.4
8	99.3	0.45	76.21	11.8
12	107.1	0.58	73.21	10.10

#### 3.3.2. Encapsulation efficiency (EE) and loading capacity (LC)

The EE of the configured TS-SLN at 25 °C/60% RH ranged from 88 to 86.4%, whereas the LC ranged from 13.3% to 12.2%. Whereas the Tables 1 and 2 showed the EE and LC of an optimized TS-SLN 88–73.21% and 13.3–10.10% respectively. Furthermore, at 25 °C/60% RH, the amount of TS trapped in SLNs remained constant or non-significant over 12 weeks. Whereas the EE and LC of the said formulation revealed a significant decrease at the storage temperatures of 40 °C/75% RH, which may link with the polymorphic lipid forms (Üner, 2006).

### 3.4. Effect on systolic blood pressure (SBP)

**Figure 3.**
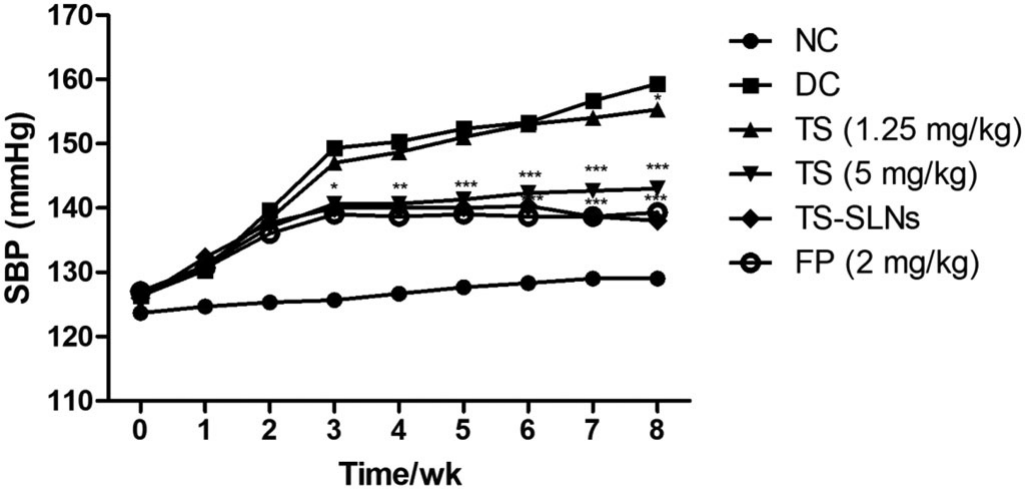
Exhibited the systolic blood pressure (SBP) in the normal and experimental group during the 8 weeks. Tail cuff method was used for the estimation of SBP. Data are presented as the mean ± SD, *p < .05, **p < .01 and ***p < .001.

Figure 3 showed the systolic blood pressure of normal and experimental mice. The normal group exhibited no difference in the SBP level throughout the experimental period. Disease control group mice showed increased SBP until the end of the experimental study. Daily administration of thiopental sodium significantly (*p* < .001) reduced the SBP at dose-dependently. Fosinopril also decreased SBP throughout the experimental period.

### 3.5. Effect on hemodynamics

**Figure 4.**
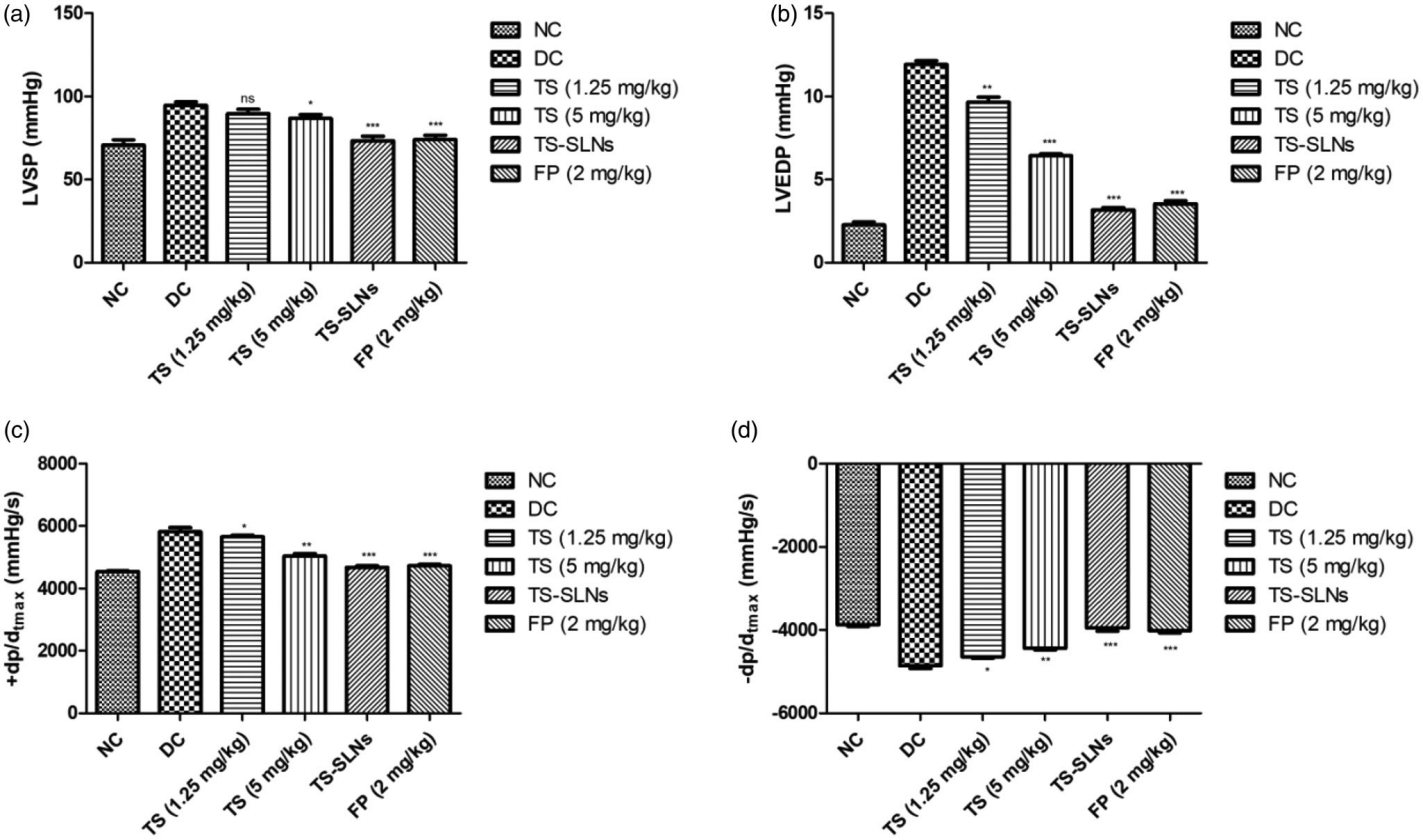
Exhibited the level of hemodynamics parameters in the normal and experimental group during the 8 weeks. (a) LVSP, (b) LVEDP, (c) +*dp*/*dt*_max_ and (d) −*dp*/*dt*_max_ Date are presented as the mean ± SD, *p < .05, **p < .01 and ***p < .001.

For the estimation of the effect of thiopental sodium on the hemodynamics, the LV function was performed at the end of the experimental study. Figure 4 shows the increased level of cardiac parameters such as LVSP, LVEDP, +*dp*/*dt*_max_ and −*dp*/*dt*_max_ in the disease control group mice. Thiopental sodium treatment significantly (*p* < .001) down-regulated the cardiac parameters such as LVSP, LVEDP, +*dp*/*dt*_max_ and −*dp*/*dt*_max_ compared to disease control. A similar result was observed in the positive control (fosinopril) treated group mice.

### 3.6. Effect on aortic remodeling

**Figure 5.**
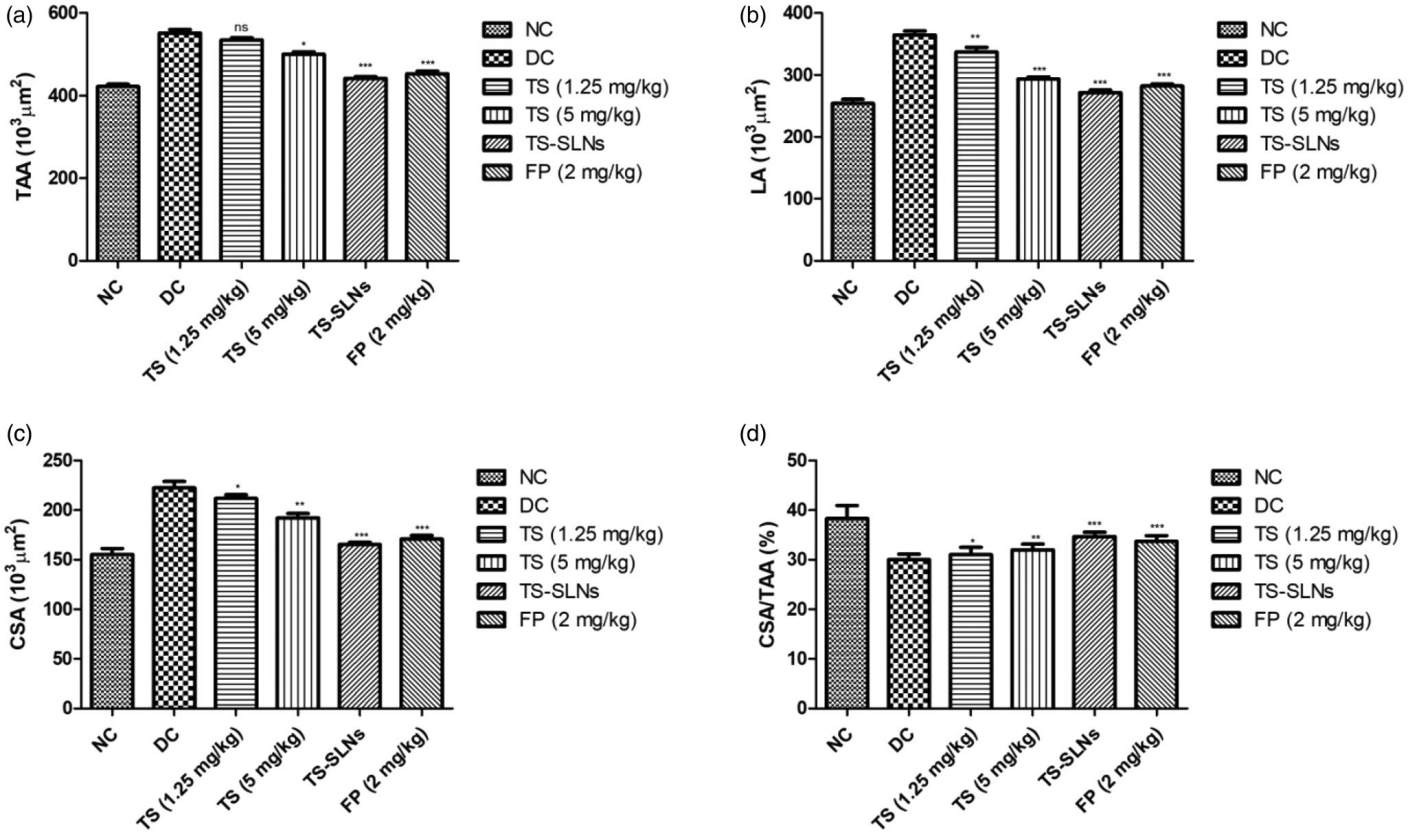
Exhibited the level of cardiac remodeling parameters in the normal and experimental group during the 8 weeks. (a) TAA, (b) LA, (c) CSA and (d) CSA/TAA. Data are presented as the mean ± SD, *p < .05, **p < .01 and ***p < .001.

Figure 5 demonstrated the effect of treated and untreated mice on the aortic remodeling parameters. Aortic parameters such as total aorta area (TAA), LA, cross-sectional area (CSA) increased and CSA/TAA ratio decreased in the disease control group mice compared to normal control mice. Thiopental sodium significantly reduced the level of TAA, LA CSA and increased the ration of CSA/TAA compared to disease control group mice. A similar result was observed in the fosinopril treated group mice.

**Figure 6.**
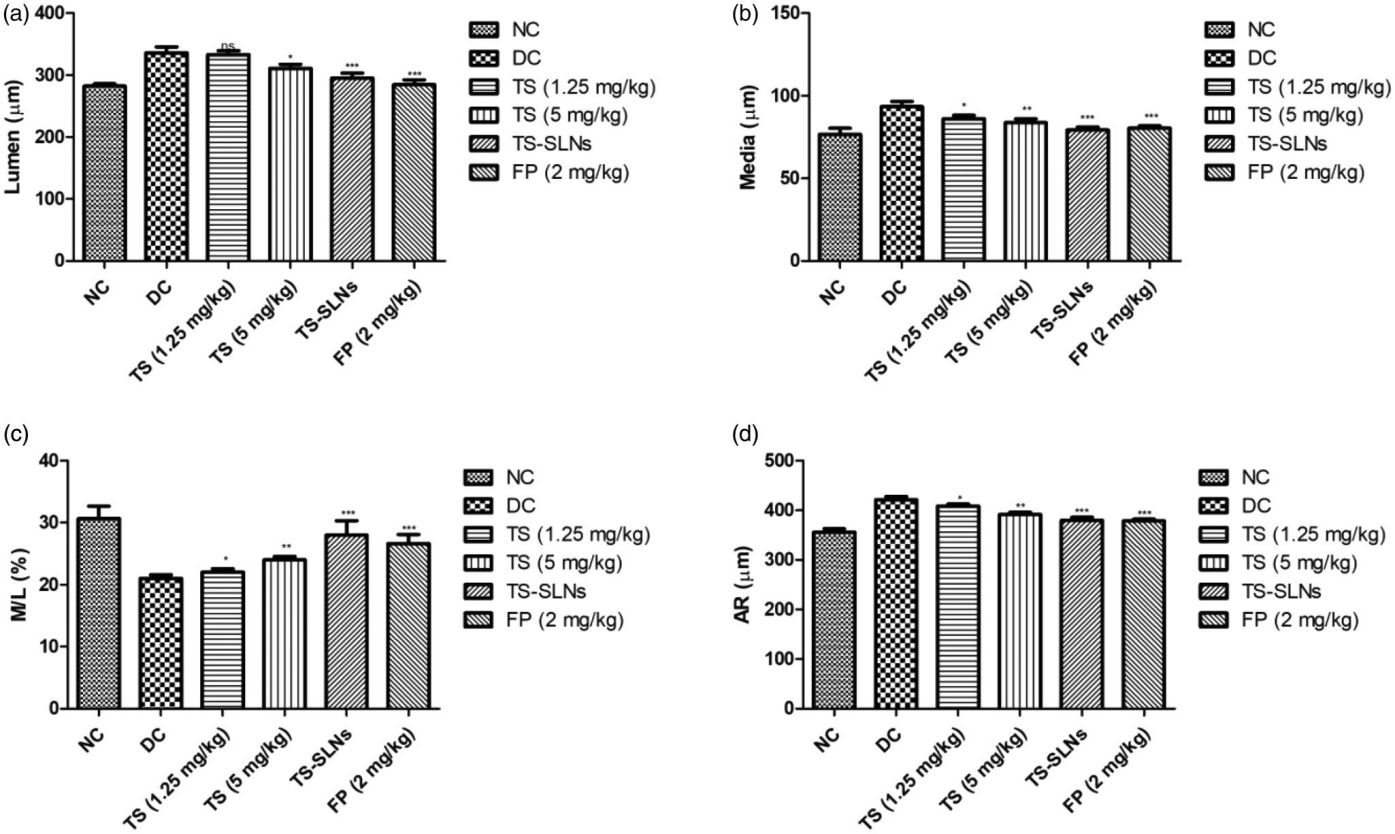
Exhibited the level of aortic parameters in the normal and experimental group during the 8 weeks. (a) Lumen, (b) Media, (c) M/L and (d) AR. Data are presented as the mean ± SD, *p < .05, **p < .01 and ***p < .001.

Figure 6 shows the level of lumen, media, aorta radius (AR) and ratio of media/lumen. Normal control group mice showed the normal level at the end of the experimental study. Disease control group mice exhibited the increased level of lumen, media, aorta radius and decreased the ratio of media/lumen. Thiopental sodium significantly (*p* < .001) down-regulated the level of lumen, media, aorta radius and up-regulation of the ratio of media/lumen.

### 3.7. Effect of thiopental sodium on L-arginine, tetrahydrobiopterin (BH_4_), NO and ET-1

**Figure 7.**
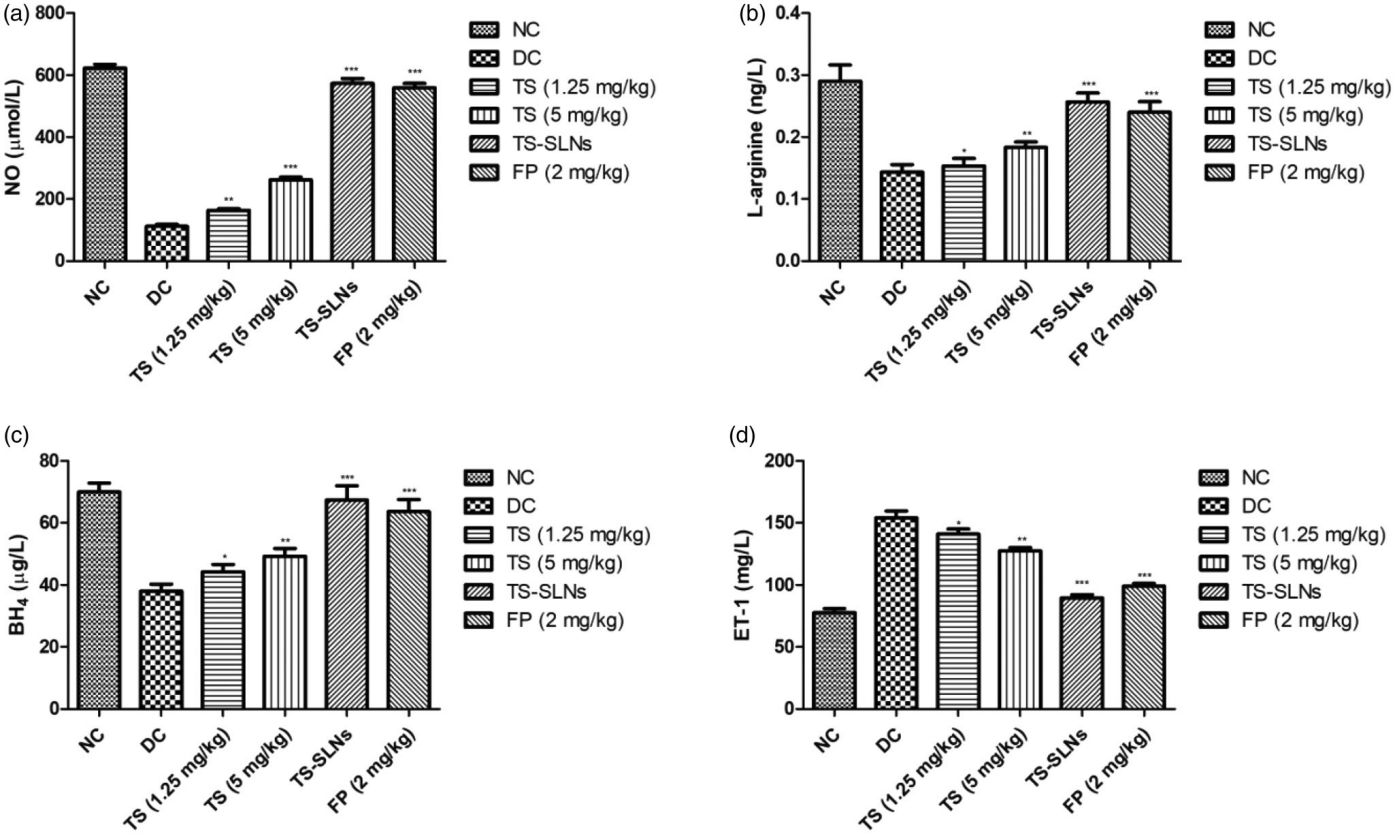
Exhibited the level of biochemical parameters in the normal and experimental group during the 8 weeks. (a) NO, (b) L-arginine, (c) BH_4_ and (d) ET-1. Data are presented as the mean ± SD, *p < .05, **p < .01 and ***p < .001.

During the cardiac dysfunction reduced the level of NO, BH_4_, L-arginine and increased the level of ET-1. Disease control group mice showed a similar result compared to normal control mice. Thiopental sodium received mice showed the increased level of NO, BH_4_, L-arginine and dose-dependently decreased the level of ET-1 (Figure 7). A similar result was found in the Fosinopril treated group mice.

### 3.8. Effect on antioxidant parameters

**Figure 8.**
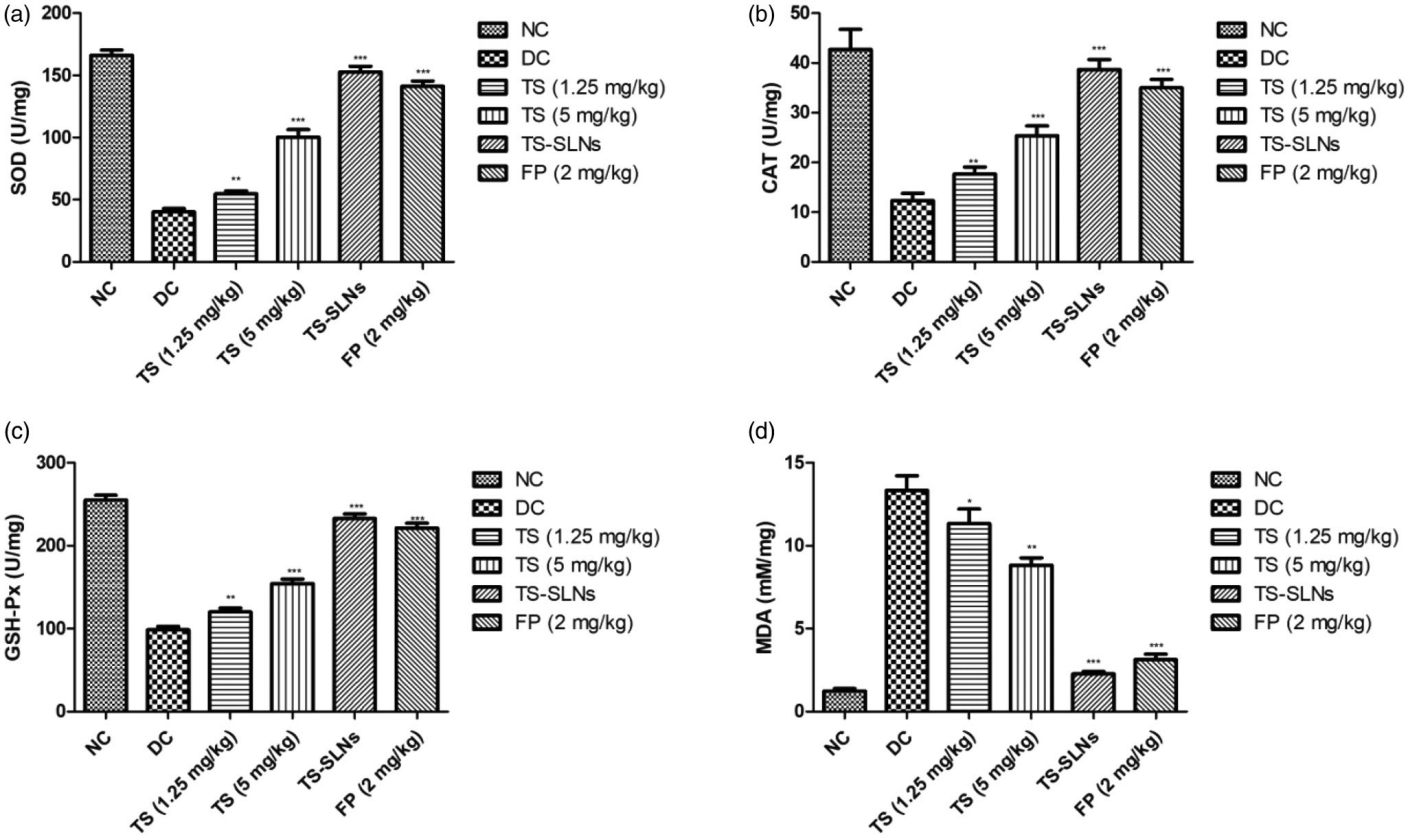
Exhibited the level of antioxidant parameters in the normal and experimental group during the 8 weeks. (a) SOD, (b) CAT, (c) GSH-Px and (d) MDA. Data are presented as the mean ± SD, *p < .05, **p < .01 and ***p < .001.

Figure 8 exhibited the effect of thiopental sodium on the level of antioxidant parameters of experimental mice. Disease control group mice showed the increased level of MDA and reduced level of SOD, GSH-Px and CAT as compared to without treated mice. Dose dependently treatment of thiopental sodium significantly (*p* < .001) down-regulated the MDA levels and up-regulated the SOD, GSH-Px and CAT level compared to disease control mice.

### 3.9. Effect on apoptosis marker

**Figure 9.**
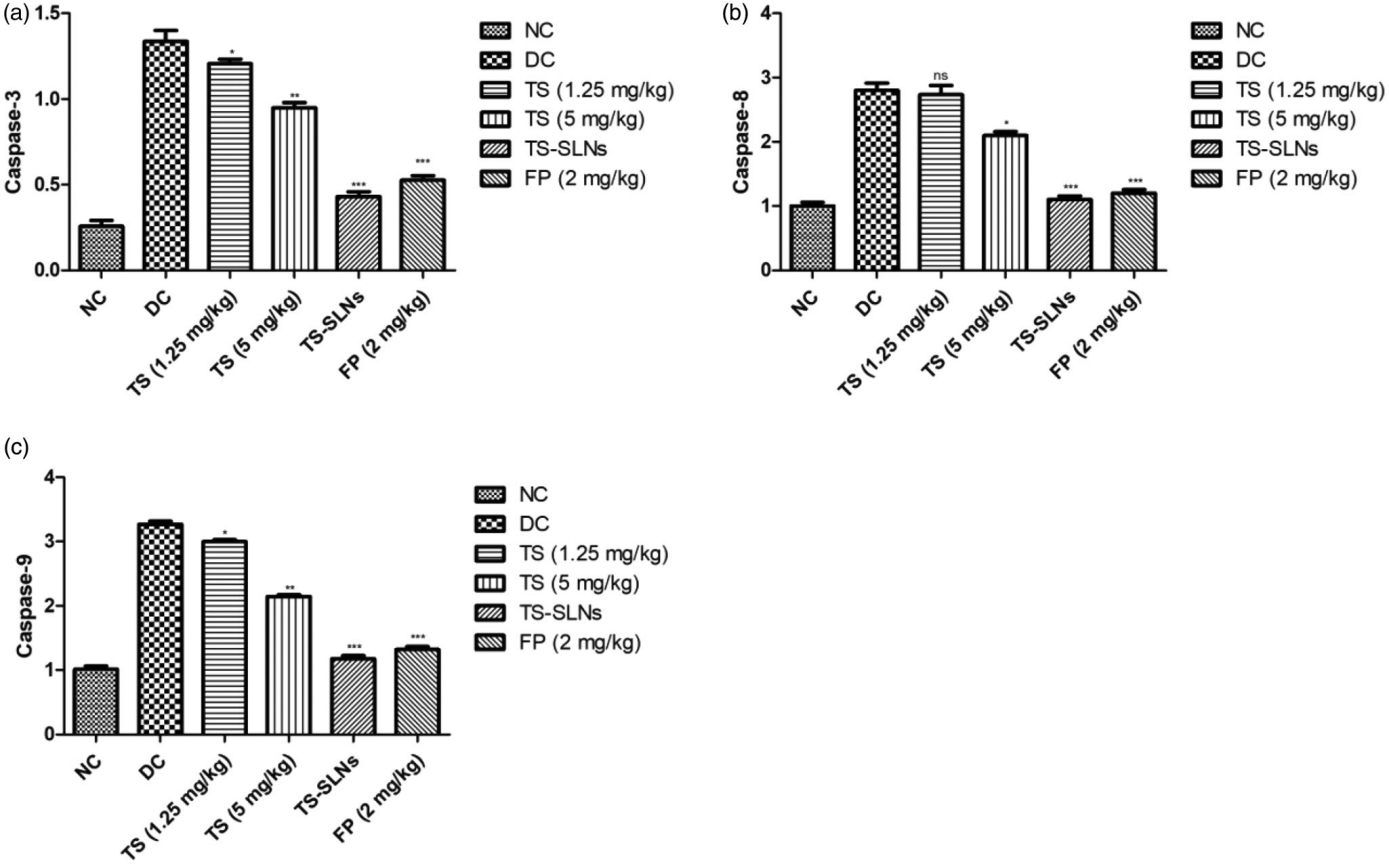
Exhibited the level of apoptosis marker in the normal and experimental group during the 8 weeks. (a) caspase-3, (b) caspase-8 and (c) caspase-9. Data are presented as the mean ± SD, *p < .05, **p < .01 and ***p < .001.

Apoptosis markers such as caspase-3, caspase-8 and caspase-9 were estimated at the end of the experimental study. Figure 9 showed the increased level of apoptosis markers such as caspase-3, caspase-8 and caspase-9 in the disease control group mice and dose-dependently treatment of thiopental sodium significantly (*p* < .001) down-regulated the level of apoptosis markers such as caspase-3, caspase-8 and caspase-9. A similar result was observed in the fosinopril treated group mice.

### 3.10. Effect on pro-inflammatory cytokine and inflammatory mediators

**Figure 10.**
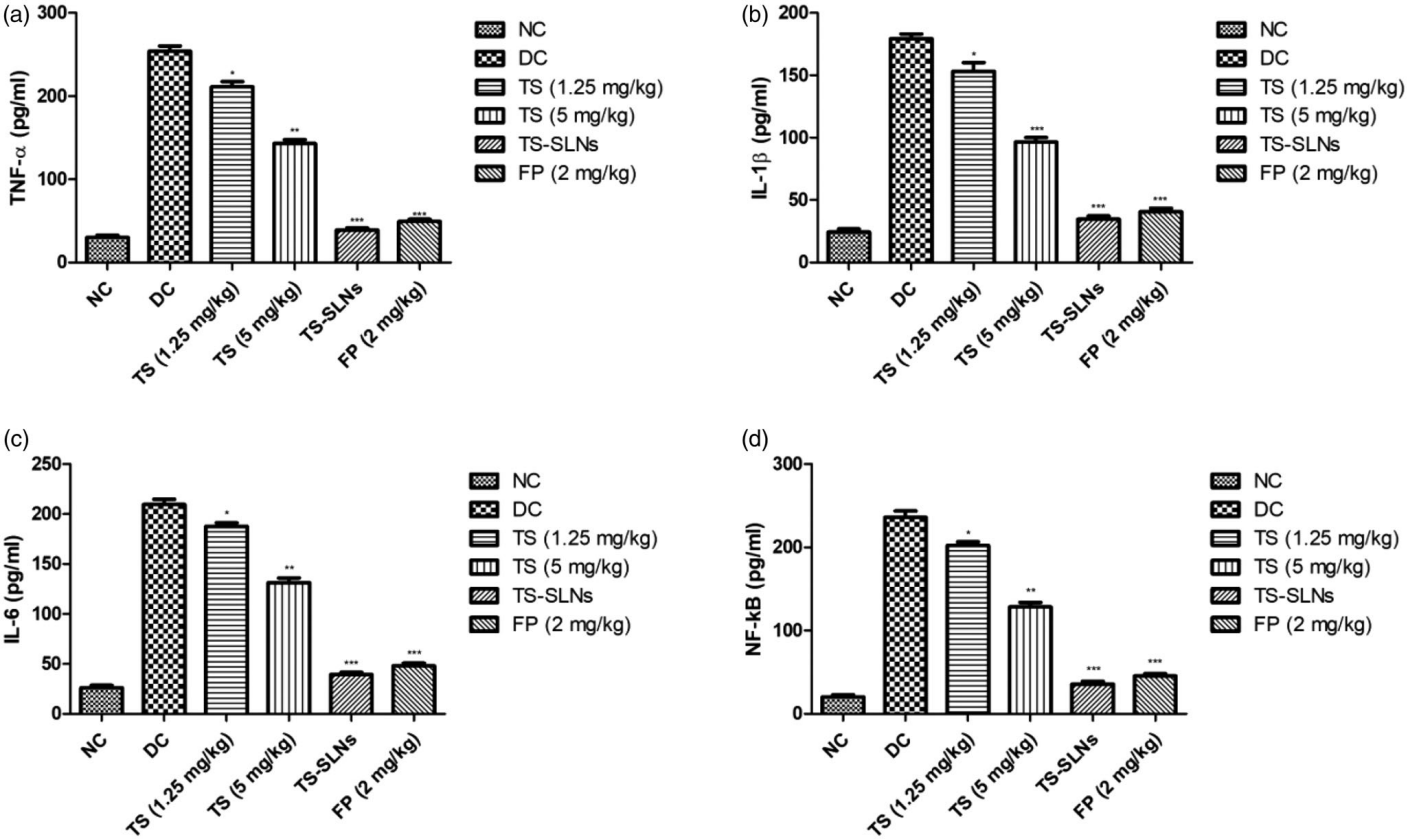
Exhibited the level of pro-inflammatory cytokines and inflammatory parameters in the normal and experimental group during the 8 weeks. (a) TNF-α, (b) IL-1β, (c) IL-6 and (d) NF-kB. Data are presented as the mean ± SD, *p < .05, **p < .01 and ***p < .001.

Pro-inflammatory cytokines such as TNF-α, IL-1β and IL-6 and inflammatory mediators like NF-kB were considerably boosted during the cardiac disease and similar momentum was observed in the disease control group mice. Thiopental sodium significantly (*p* < .001) suppressed the level of pro-inflammatory cytokines and inflammatory mediators compared to the disease control group mice (Figure 10).

### 3.11. Effect on p38-MAPK

**Figure 11.**
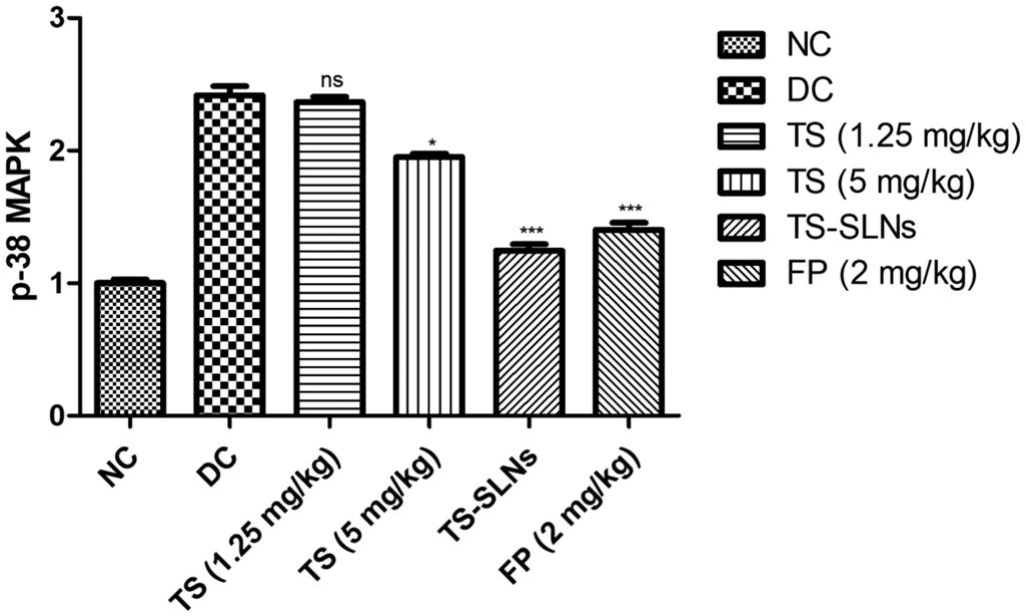
Exhibited the level of p-38 MAPK parameter in the normal and experimental group during the 8 weeks. Data are presented as the mean ± SD, *p < .05, **p < .01 and ***p < .001.

The level of p38-MAPK considerably boosted during the cardiac disease and similar result was observed in the disease control group mice. Thiopental sodium significantly (*p* < .001) reduced the level of p38-MAPK compared to disease control mice (Figure 11).

## 4. Discussion

In this study, developed TS-SLNs and explored the efficacy of same by oral administration in prevention of obesity-induced Cardiac dysfunction and Cardiac hypertrophy in mice model. TS-SLNs founds significant improvement in the availability of TSand demonstrated the potential effect againstobesity-induced Cardiac dysfunction and Cardiac hypertrophy (OICD-CH) via anti-inflammatory mechanisms. In OICD-CH, TS acted as cardiac protective potential, but it possesses several hiccups, which may minimize by the dose and effective delivery of TS by loading into SLNs. The unique features of TS-SLN include small particle size, long-term release properties, biocompatibility and biodegradability as an attractive drug delivery to OICD-CH. Hot-homogenization and solvent diffusion method as stated throughout literature reports with suitable modifications (Rahman et al., 2019). The selection of amount excipients as Compritol 888 ATO (i.e. 250 mg) and Phospholipid 90 G (PL90G) (60 mg) were chosen as the solid lipid and co-surfactant. Further addition of 3% w/v of aqueous solution of Tween 80, homogenization speed at 10,000 rpm, stirring speed and time have great impact on particle size, entrapment efficiency and loading capacity. We also observed some difference in the size obtained by TEM with Dynamic Light Dispersion (DLS). These variations can be attributable to the determination of the solid state SLN by TEM, but the hydrodynamic diameters of the mixture in solution can be determined in case of a zetasizer (Nayak et al., 2010; Ekambaram & Abdul Hasan Sathali, 2011). Therefore, our overall results align with and support the findings of other researchers (Üner, 2006; Nayak et al., 2010). Further, TS-SLNs exhibited biphasic pattern of drug release within the first 4 h time span and maximum drug release rapidly >58% within the said time. While the remaining amount of drug release was observed within next 8 h.In addition, this may attribute to the existence of SLN encapsulated TS following a drug release mechanism through the mechanism of surface erosion.

Previous research suggests that LVH enhanced cardiovascular diseases related mortality and morbidity (Lang et al., 2018; Wang et al., 2019). It is proofed that the CVD risk factors are linked with the expansion of LVH. Another parameter of CVD is hypertension and its mostly determinants of LVH in the general populations. Recently clinical and epidemiological investigations exhibited that the hypotension is the strong predictor of LVH especially eccentric LVH (Stef Á Nsson et al., 2014; Schultz et al., 2017). In the meantime, increased BP level plays a crucial role in activating LV myocardial expansion via chronic hemodynamic overload and enhanced central pressure. Primary hypertension disease in which environmental and genetic factors interact, with the fat intake being a significant environmental marker (Lang et al., 2018; Wang et al., 2019). Various studies on the animals and epidemiological surveys have exhibited a positive correlation between the BP level and fat intakes. Additionally inducing an increase in BP, high fat intake can also induce LVH, heart failure, and cardiovascular accidents. In the current experimental study, the result demonstrated that the regular adsorption of high fat diet resultant enhance in LVH and SBP in the experimental rodents, evidenced via the worsening of cardiomyocyte CSA, cardiac hemodynamics, media thickness, longitudinal diameter and HW/BW ratio, treatment with TS-SLNs markedly improved these pathophysiological alterations (Siti et al., 2015; Lang et al., 2018; Wang et al., 2019).

Excessive ROS produced during oxidative stress is well known, resulting in damage to the antioxidant protection system intercellular. Start accumulation of ROS during oxidative stress, triggers membrane protein, DNA mutation, enzyme denaturation and lipid peroxidation, causing irregular cell activity and eventually irreversible cell harm or death (Black & Garbutt, 2002; Siti et al., 2015). Over the past few years, evidence indicates that oxidative stress is a major cause of irregular cardiovascular system function and structure, and ROS produced during oxidative stress is strongly linked to several incidences and development of CVDs (Black & Garbutt, 2002; Madamanchi et al., 2005). Previous literature indicates that the oxidative stress injury in high fat mediated hypertensive mice, which is expressed mainly by enhanced peroxidase, lipid peroxidative, superoxide anion production, and oxidase activity of the nicotinamide adenine dinucleotide phosphate (NADPH) (Zheng & Tang, 2016; Geng et al., 2019). MDA is the end product of lipid peroxidation. First-line endogenous antioxidant, such as SOD and CAT, are known to be the essential antioxidant enzymes in the myocardial tissue, which plays a significant role in preserving myocardial cells from oxidation by converting the superoxide anions (O_2_) into the hydrogen peroxide (H_2_O_2_) (Du et al., 2020). Also, estimating the level of MDA, CAT, and SOD in the myocardium can indicate the level of ROS and degree of LPO inside the body (Zheng & Tang, 2016; Du et al., 2020). Throughout this experimental research, we found the increased level of MDA and reduced levels of SOD, CAT in the disease control group and thiopental sodium and fosinopril significantly (*p* < .001) decreased the level of MDA and increased the level of CAT, SOD in the serum. The outcome suggests that TS-SLNs will impose antioxidant ability and can the level of oxidative stress throughout the body.

The inflammatory reaction is playing a significant mechanism of essential endothelial damage pathway, which plays a significant role in the coronary disease pathology (Arozal et al., 2011; Wu et al., 2012). Prior work indicates that the C-reactive protein (CRP) is an inflammatory precursor, directly linked in the later stage of hypertension to coronary remodeling (Black et al., 2004). CRP induces the vascular endothelium injury, resultant in decreased production of vasodilators. When inflammation occurs, mononuclear, B lymphocyte and T lymphocytes are activated to release the huge amount of IL-6 and TNF-α. TNF-α is mediated with the various processes include inflammation, apoptosis, cell growth and survival (Kaptoge et al., 2012; Shrivastava et al., 2015). During cardiovascular disease, TNF-α can quickly enhance the endothelial cell adhesion factors, expand the accumulation of the inflammatory cells, activate the endothelial cells and finally start the secretion of inflammatory mediators into the serum. It also affected the biological and morphological character of endothelial cells (Ridker & Silvertown, 2008; Poledne & Králová Lesná, 2018). TNF-α regulates the remodeling and injury of endothelial cells via the NF-kB signaling pathway. Previous studies suggest a relationship between the endothelial tissue and inflammation. Injury into the endothelium is showing the effect on the quantity and functions of endothelial cells, the decrease nitric oxide bioavailability and synthesis and boosts the ROS production (Berg & Scherer, 2005; Kaptoge et al., 2012). It is well documented that the expansion of TNF-α during heart disease especially cardiac hypertrophy via activation of inflammatory pathway and apoptotic along with the suppression of mitochondrial electron transport chain complexes. During the cardiac disease, increase the synthesis of cytokines especially TNF-α in the heart tissue by triggering the NF-kB. The suppression of anti-apoptotic along with the cytokines such as TNF-α in the cardiac myocytes showed a beneficial effect on cardiac remodeling. IL-10, potent cytokines and contribute to the reduction of cardiomyocyte apoptosis and survival of cardiac cells (Berg & Scherer, 2005; Ridker & Silvertown, 2008; Poledne & Králová Lesná, 2018). TS-SLNs considerably altered the level of cytokines and inflammatory mediators via reduce the cytokines and apoptosis in cardiac and suggesting the anti-inflammatory effect.

It is well proved that p38 MAPKs play a significant role in the cellular process including apoptosis, cell contraction, inflammatory reaction, and cell growth and the expansion of CVD, such as cardiac hypertrophy (Graves et al., 1996). Previous experiments showed that cardiac myopathy triggered apoptosis in cardiac myocytes is mediated through stimulation of p38 MAPK and dysregulated apoptotic machinery Activation of p38 MAPK is typically accomplished via its phosphorylation and is associated with the initiation and development of pathological hypertrophy and cell death (Kim & Choi, 2010; Fisk et al., 2014). Disease control group rats exhibited the increased level of p38 MAPKs and boost the inflammatory reaction and thiopental sodium reduced the level of p38 MAPKs and suggesting the cardio-protective effect via inhibition of inflammatory pathway (Muslin, 2008; Fisk et al., 2014). To investigate whether the anti-apoptotic effect of thiopental sodium is related via p38 MAPK, we scrutinized the apoptosis marker. Disease control rats exhibited an increased level of Bax, caspase-3, caspase-8, caspase-9 and reduce the level of Bcl-2 and suggesting increased apoptosis. TS-SLNs significantly (*p* < .001) reduced the level of Bax, caspase-3, caspase-8, caspase-9 and increase the level of Bcl-2 and suggesting the anti-apoptosis effects.

## 5. Conclusion

Therefore, we hypothesized that solid lipid nanoparticle of thiopental sodium is capable of reducing cardiac impairment, hypotension and cardiac hypertrophy in cardiac dysfunction caused by obesity. The cardiac remodeling function, aortic function, apoptosis marker, oxidative stress parameter and inflammatory parameters were greatly altered by the thiopental sodium treatment. Besides, more research must determine the fundamental mechanism and potential therapeutic effects.
